# Treatment of Chronic Diseases of the Heart

**Published:** 1852-11

**Authors:** 


Translated from the French.

*Treatment of Chronic Diseases of the Heart.*

(CONCLUDED FROM LAST NUMBER.)

*Narcotics.* One is often tempted to give to patients affected with asthma and a severe suffering, which makes them desire death, a narcotic preparation, opium, the salts of morphine or belladonna. Hope says that opium united to digitalis has been serviceable in his hands in overcoming this morbid state. In proportion as the symptoms are more nervous than vascular, they may be moderated by narcotics and anti-spasmodics, and in this secondary manner the cardiac and pulmonary circulation may be acted upon.

If we except this special indication, I have seen only bad results from the use of opium. One may, without doubt, quiet the nervous system, and the patient for a time realize less sensibly his sad condition; but it is only for a very short time, and purchased at the price of frightful dreams, a painful awakening and a suffering more intense than before. Besides, opium augments the cardiac and pulmonary congestion, enfeebles innervation and muscular irritability, and assists in increasing still more the cyanosis, the cerebral congestion, and that which alrerdy exists in the other organs.

*Tonics and excitants.* I have already alluded to the embarrassment of the physician called to treat an affection of the heart, and compelled to choose between debilitants and tonics. An opinion which at the present time has many adherents in Germany, and even in France, attributes great efficacy to the tonic treatment and especially quinia, preparations of iron, bitters, and a generous diet. This treatment is not adapted to all cases; it is, however, more frequently applicable than the debilitating treatment, and is free from many of its dangerous consequences.

In order to derive from tonics the greatest benefit, they should be administered in cases in which there is present, one or more of the following morbid conditions :

1st. If the heart is already affected with passive aneurism, and the venous system of the cephalic extremity congested, we may seek to stimulate the heart by substances such as quinia, aromatic tinctures of cinnamon, of menthæ, wine, brandy, ether, acetate of ammonia, and coffee ; which I have found beneficial in some cases almost desperate.

2d. These therapeutic agents ought to be liberally employed, in cases where there are chronic lesions of the respiratory passages, as catarrh, emphysema, or pulmonary tubercles ; they may be associated with expectorants, which act admirably in exciting the bronchial capillaries ; such are the preparations of Scilla, Kermes, Tartrate of Antimony, and Ipecac, given in nauseating doses, Sulphur, Nitrate of Potassa, Balsam of Tolou, Benzoin, Pitch, Turpentine, Morton's Pills, &c.

3d. The preference should be given to bitter tonics, to preparations of Iron, Quinine, and a tonic and stimulant diet, when there is reason to fear debility ; their use should be still more strongly enjoined, in cases of symptomatic anæmia.

4th. Albuminuria, which may be called congestional, to distinguish its true nature, can be advantageously modified by the preceding medication.

5th. Lastly, in feeble, dropsical subjects, the stimulation of the vascular system ought to be but rarely tried, and then, aided by blisters placed over the precordial region, the sides of the chest, the hypochondriac regions, and the inferior extremities. By these means, the action of the heart, in phlegmatic subjects, who are not too excitable, may sometimes be restored. It must not be forgotten, that life having been diminished in the capillaries, gangrene easily takes possession upon skin denuded, or distended with serum ; we should, therefore, have recourse to blisters only in cases where other stimulating agents have failed. We may substitute for them, alkaline, saponaceous, or sulphurous lotions, frictions, with aromatic liquors, &c. They will be injurious where the skin is strongly distended with dropsical effusions.

The dietetic regimen to which the patient should be confined, is an important part of the tonic treatment. I have nothing in particular to say, in regard to it, except to advise not to proscribe wine or moderate exercise, which appear to me rather useful than injurious. I have seen unfortunate aneurismatics come into our hospitals, worn down by suffering, by hardship, and often by intemperance; but relieved by the aid of vinous drinks and coffee, and a generous, reparative diet.

*Anti-spasmodics.* With the preceding medication may be associated the anti-spasmodics, such as ether, musk, valerian, succinate of ammonia, for the purpose of modifying, or even arresting the asthma, the precordial anxiety, the palpitation, and the restless wakefulness which accompany pulmonary congestion.

*Medicines adapted to excite the capillary circulation in the organs.* The heart is often unable to impel the blood in the parenchyma, and in spite of the preceding treatment, the capillaries remain congested. These congestions may be overcome, either directly, by blood-letting, as I have already said, or by exciting the secretion of products proper to the engorged tissue. It is thus that by the aid of diaphoretics, and the sweating properly conducted, the cutaneous capillaries may be disengorged; that by drastic purgatives, as elaterium, colocynth, and bryona, secretions, often abundant and salutary, of serum, mucus, or bile of the intestines may be excited, that with diuretics producing copious urinary secretions, the renal congestion may be sometimes reduced. The bronchial capillaries are more difficult to be relieved.

Ipecac. pulv. Dov. emetics and expectorants, as already indicated, may be administered. Vomiting by the contraction of the diaphragm, and the disturbance which it induces, excites favorably the capillaries. I will even say that this medication is the only one by which we may act directly upon the pulmonary circulation. It is only necessary to select the cases, and not to prescribe this dangerous treatment for patients already very much enfeebled, and in the latter stages of the cardiac affection.

From our examination of the principal methods of treatment, it results; 1st. That to correct the feebleness of cardial propulsion, the tonic and stimulating medication should, in general, be preferred to blood-letting, digitalis, and narcotics. 2d. That it is necessary to restore to the blood its physiological properties, by tonics, and ferruginous preparations. 3d. That in addition to the above, treatment should be directed to the bronchial, renal, hepatic, intestinal and cutaneous circulation. The treatment indicated by Senac, Corvisart, and Laennec, is still that which offers the most chances of success.

I have had no other end in view, in writing this essay, than to localize, in a small number of cases, the origin of the various symptoms of diseases of the heart; and then to point out, along with the therapeutical indications derived from the pathological physiology, the agents the most capable of meeting them. I have wished, also, to furnish to the practitioner, a guide to enable him to distinguish, in symptoms, that which is mechanical and hydraulic on the one part, vital and dynamic on the other; and, finally, to point out the danger and utility of blood-letting, digitalis, and a few other therapeutic agents. J.

